# Valorization of Beetroot By-Products for Producing Value-Added Third Generation Snacks

**DOI:** 10.3390/foods12010176

**Published:** 2023-01-01

**Authors:** Marta Igual, Faustine Moreau, Purificación García-Segovia, Javier Martínez-Monzó

**Affiliations:** 1 Food Technology Department, Universitat Politècnica de València, Camino de Vera s/n, 46021 Valencia, Spain; 2 Institut Agro Dijon, 26, Boulevard Docteur Petitjean, 21000 Dijon, France

**Keywords:** upcycling, extrusion, total phenols, antioxidant capacity, physicochemical properties, betalains

## Abstract

Food waste is becoming a growing and important concern at both local and global levels. One-third of all food production is lost or wasted globally. It is necessary to look for alternatives that allow the use of agri-food waste or byproducts and that can provide value to other foodstuffs. The utilization of beetroot byproducts for producing value-added third generation (3G) snacks was the main aim of this work. These snacks are obtained by indirect expansion by extrusion and later heat expansion. In order to achieve this aim, a corn grits base was used and the influence of water content and beetroot byproduct content effect was studied on expansion kinetics by microwave energy and on texture, colour, extrusion parameters and bioactive compounds of expanded 3G snacks. The microwave expansion kinetics study determined the appropriate time to expand the formulations studied. Samples with higher water content in the mixtures needed more expansion time. In terms of expansion, all samples presented acceptable values; however, samples with 25% water in the mixtures showed better results. Furthermore, these snacks showed more crunchiness and less hardness. Beetroot byproduct incorporation provided additional functional value to the snacks. The betalains and phenols contained in the beetroot byproduct were presented in the expanded snacks and increased the antioxidant capacity of the snacks. With this study, it can be recommended to use 25% water content and 10% beetroot byproduct in corn mixture to obtain a third-generation snack with added value.

## 1. Introduction

Food waste is becoming a growing and significant concern both locally and globally [1,2]. According to the Food and Agriculture Organization of the United Nations, one third of all food production is lost or wasted globally, which is equivalent to 1.3 billion tons of food produced for human consumption wasted per year, with an economic loss of EUR 800 billion [3]. Approximately 44–47% of this is fruit, vegetables, fish, and meat that is produced each year and wasted [4].

The composition and great availability of food waste have contributed to the seeking of alternatives to its disposal.

Fruits and vegetables, in addition to their nutritional value, are generally recognized as important sources for a wide array of phytochemicals, which individually, or in combination, may benefit human health, and thus are considered bioactive compounds [5].

Beetroot (*Beta vulgaris* L.) is a dicotyledonous plant of the *Chenopodiaceae* family. It is mainly grown for its roots, intended for various uses: sugar beet (sugar production), fodder beet (animal feed) and vegetable beet (red beet, human consumption). The latter is consumed by many populations, in juices, salads or accompaniments, for example, and is used for the production of a food colouring authorized within the European Union (E162: beet red) [6,7]. Beetroot contains flavonoids, carotenoids, minerals, vitamins and betalains, water-soluble pigments such as betacyanins (red-violet), and betaxanthins (yellow-orange) that provide numerous nutritional and health benefits [8]. Studies have shown that beetroot is an important source of phytochemicals beneficial to health [9].

**Figure 1:**
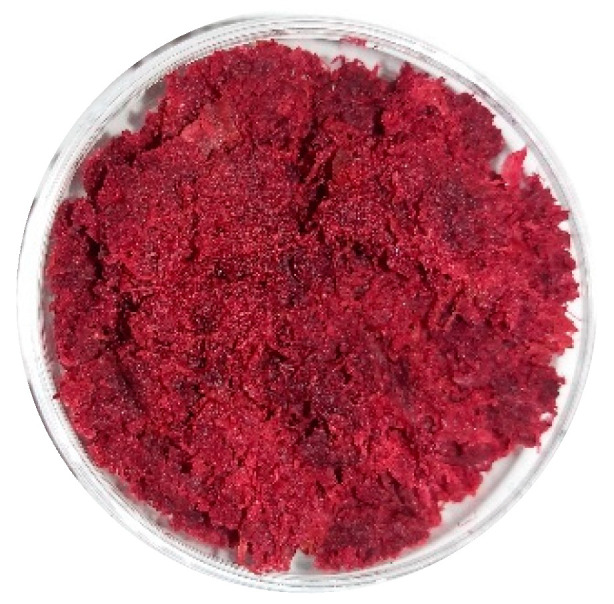
Appearance of beetroot byproduct recovered from the process of obtaining beetroot juice by liquefying.

However, in industrial processes the beetroot byproduct is very large. Approximately 40% of the beetroot is waste in the process of obtaining liquefied beetroot to be atomised. The liquefied beetroots are usually atomised to obtain natural colourants. The byproduct produced from obtaining the juice is a fibrous mass with a typical purple coloration of beetroot, as can been observed in Figure 1. The uses of this waste are usually either animal feed or biofuel.

Currently, the eating habits of populations have evolved towards healthier eating [10]. There is also an increase in snacking as food consumption between main meals, according to different studies [11,12,13]. Therefore, the proposal to obtain healthy snacks combines the two trends. In addition, if beetroot byproduct is used in formulation, a residue from the food industry is incorporated, valuing it, providing added value to the final product, and reducing food waste. In this way, the circular economy is promoted as recommended by the FAO (Food and Agriculture Organization) in SDG 12 (Sustainable Development Goals) and contributes to achieving the 2030 goal of reducing food losses in production chains.

In contrast to other calorie-rich snacks, the extrusion process allows for the production of low-calorie density snacks [14].

Extrusion cooking technology is considered a versatile and modern technology with high-temperature, short-time food operation. Cooking food at high temperatures, in a matter of seconds, often has favorable effects on maintaining the properties of food components and active ingredients, while drastically reducing or completely eliminating micro-organisms present in the starting materials. Therefore, the final extruded food product, with a low moisture content, is considered a shelf-stable product [15]. Depending on the formulation and processing conditions, the extruded product can undergo direct or indirect expansion. Direct expansion is the most common form [16], and refers to the immediate expansion of the product as it exits the extruder die, resulting in a second generation snack (2G Snacks). In contrast, indirect expansion occurs after extrusion, in a separate thermal device, providing a third-generation expanded snack (3G Snacks) from an unexpanded pellet produced in the extruder.

Pellets called 3G snacks are formed via extrusion at high moisture content, moderate temperature, and are dried, allowing the preservation of thermally sensitive ingredients [17]. The expansion process of these pellets can be performed by a deep-fryer, oven or microwave in order to achieve a crispy texture. This methodology provides an alternative in terms of generating nutritious food, and it also reduces export costs in which the volume usually raises the final sale costs since 3G pellets take up less space than expanded snacks. These snacks are usually made from corn.

For expanding pellets, in classic heating the material absorbs energy from thermal gradients by convection, conduction and radiation. In contrast, microwave energy (MW) interacts directly with the material molecules, resulting in a conversion of energy into heat instead of heat transfer. Therefore, MW can reduce processing times and energy consumption, thus improving energy efficiency. When MW is applied to starch-based materials, the factor that initiates the heating process is the water content [18]. Consequently, as the starch matrix heats up and the temperature increases, the water molecules become a superheated steam, creating a high local pressure. When the temperature is high enough, the pellet matrix undergoes a phase transition from a glassy to a rubbery state and, combined with the high pressure of the superheated steam, expands [19]. If microwave heating is interrupted at the right time, the matrix returns to the glassy state and the foamed air spaces are retained due to the mechanical resistance of the matrix in the glassy state, generating a crispy texture that attracts consumers [20].

The utilization of beetroot byproducts for producing value-added third generation snacks was the main aim of this work. Consequently, the specific objectives of this research were (1) to examine the influence of water and beetroot byproduct content of the mixtures on expansion kinetics by microwave energy, and (2) to evaluate the effect of water and beetroot byproduct content of the mixtures on texture, colour, extrusion parameters and betalains, total phenols and antioxidant capacity of expanded 3G snacks.

## 2. Materials and Methods

### 2.1. Raw Materials

Corn grits were provided by Maicerías Españolas S.L. (València, Spain). Beetroot byproduct was recovered from the process of obtaining beetroot juice by liquefying in an electrical food processor (DeLonghi, Barcelona, Spain) for 10 s [21], similar to the industrial process.

### 2.2. Beetroot By-Product Powder Manufacturing

Beetroot byproduct was placed on an aluminum plate and stored at −45 °C (Vertical Freezer, CVF450/45, Ing. Climas, Barcelona, Spain) for 24 h. The samples were then dried in a Lioalfa-6 Lyophyliser (Telstar, Spain) at 2600 Pa and −56.6 °C for 48 h. The freeze-dried sample was ground in a grinder (Minimoka, Taurus, Lleida, Spain) to obtain a free-flowing powder.

### 2.3. Formulation and Preparation of Corn 3G Extruded Pellets

Corn grits were mixed thoroughly with water and different percentages of beetroot byproduct (5 and 10%). The water content of the mixtures for extruder feed was adjusted to 25% and 30% (wet base) by mixing continuously at medium speed in a mixer (Bosch MFQ40303, Gerlingen, Germany). In this way, the following samples were obtained: C25, 25B5 and 25B10 for the samples with 25% moisture and 0%, 5% and 10% beet byproduct, respectively, and C30, 30B5 and 30B10 for the samples with 30% moisture and 0%, 5% and 10% beet byproduct, respectively.

Corn third-generation pellets were produced with a single-screw laboratory extruder (Kompakt extruder KE 19/25; Brabender, Duisburg, Germany). The extruder had a barrel diameter of 19 mm and a length diameter ratio of 25:1. It was operated at a 3:1 compression ratio and a constant dosing speed of 20 rpm. The screw was rotated at 120 rpm. The temperatures of the barrel sections were 30, 60, 100, and 120 °C, and the nozzle diameter was 3 mm. The calculated specific mechanical energy of the corn extrusion ranged from 820 to 1082 kJ/kg and the pressure ranged between 25 and 40 bar. The extruded products were dried at 25 °C for 18 h. The dried samples were stored in polyethylene bags at room temperature (25 °C) and used for further analysis.

### 2.4. Microwave Expansion

The expansion process was performed in a microwave oven (FT339, Whirlpool Corporation, MI, USA) at 1000 W/g of emitted power. To evaluate the microwave expansion-kinetics and dehydration-kinetics of the different corn 3G extruded pellets, the water content and characteristic dimensions of pellet samples were analyzed after 10, 20, 30, 40, 50, 60, 75, and 90 s of microwave expanded application time.

#### 2.4.1. Microwave Dehydration-Kinetics

Microwave dehydration curves were obtained from the experimental water content of the samples after different process times. The drying behaviour of the pellets was described by three commonly used thin-layer drying models: Page (MR = exp (−kt^n^)), Logarithmic (MR = a exp (−kt) + c), and Midilli-Kucuk (MR = a exp (−kt^n^) + bt) [22]. In these models, MR (moisture ratio) represents the dimensionless moisture ratio as Equation (1).

(1) $$MR=\frac{M_{t}-M_{e}}{M_{0}-M_{e}}$$

*M_t_*: Water mass fraction of the sample at each drying time (g_water_/g_product_);
*M_e_*: Water mass fraction of at the end of the process (g_water_/g_product_);
*M_0_*: Water mass fraction of sample before the process (g_water_/g_product_);
*a*, *b*, *c* and *k*: Drying constants;
*n*: Drying exponent;
*t*: Microwave-drying time (h);

Water content (x_w_) was obtained by vacuum drying the samples in a vacuum oven (Vaciotem, J.P. Selecta) at 103 °C for 48 h.

#### 2.4.2. Microwave Expansion-Kinetics

The sectional expansion index (SEI) and volumetric expansion index (VEI) of extrudates were determined following the methodology described by Patil et al. [23]. The width and large of expanded pellets were measured with a digital caliper (Comecta S.A., Barcelona, Spain).

### 2.5. Analytical Determinations

#### 2.5.1. Water Activity and Water Loss

Water activity was determined using a hygrometer (AquaLab PRE, Decagon De-vices, Inc., Pullman WA, USA). Water losses for extrusion, drying and expansion were calculated from the water content of samples after these processes. The water content of samples was determined as shown in Section 2.4.1.

#### 2.5.2. Extrusion Parameters

Βulk density (ρ_b_) is considered as expansion in all directions [24]. For ρ_b_ analysis, measurements were carried out 15 times, where the diameter and height of cylinders were measured with an electronic Vernier caliper (Comecta S.A., Barcelona, Spain) and samples were weighed with a precision scale (±0.001 g) (Mettler Toledo, Greifensee, Switzerland). The porosity (ε), percentage of air volume related to the total volume was calculated from the true (ρ) and bulk (ρ_b_) densities according to García-Segovia et al. [25]. The ρ of the extruded products was determined using a helium pycnometer (AccPyc 1330, Micromeritics, Norcross, GA, USA).

To evaluate the hydration properties, the water absorption index (WAI) and water solubility index (WSI) were used. WSI and WAI were determined by the method of Singh and Smith [26] and calculated according to Uribe-Wandurraga et al. [27]. The swelling index (SWE) was measured using the bed volume technique. The bed volume was recorded and expressed as mm of swollen sample per g of dry initial sample [28].

Hygroscopicity (H_y_) was determined according Cai and Corke [29]. Samples were placed in a petri dish at 25 °C, in an airtight plastic container containing Na_2_SO_4_ saturated solution (81% relative humidity). After 7 days, each sample was weighed and the hygroscopicity (H_y_) was expressed as g of water gained per 100 g dry solids [29].

Texture properties were measured using puncture tests (TA-XT2 Texture Analyzer, Stable Micro Systems Ltd., Godalming, UK) and software (Texture Exponent, version 6.1.12.0) [30]. From a force-time curve, the area under the curve plot, which represented the work done for a time of displacement of the puncturing device, was obtained. The force-drop of each peak was also obtained, and it represented the local resistance of cell walls, and the number of peaks (N_o_) were also recorded. These parameters were used to calculate the average puncturing force (F_p_), average specific force of the structural ruptures (F_s_), the spatial frequency of structural ruptures (N_sr_), and crispness work (W_c_) according to Bouvier [31] and Igual et al. [30].

Optical properties were determined evaluating translucency and CIE*L*a*b* color coordinates that were determined from the surface reflectance spectra obtained between 400 and 700 nm, when measuring on white and black backgrounds, considering standard light source D65 and a standard observer 10° (Minolta spectrophotometer CM-3600d, Tokyo, Japan). Hue (h*), and chroma (C*) color attributes were calculated from CIE*L*a*b* color coordinates. The total color difference between the mixture before extrusion and the finished expanded product (ΔE_1_) were calculated for samples. To evaluate the total color differences between the dry pellet and the finished expanded product, ΔE_2_ was also calculated.

#### 2.5.3. Bioactive Compounds

- Betalains

The betalains (betacyanins and betaxanthins) contents were measured according to Nilsson [32], with some modifications [21]. Samples were mixed with a phosphate buffer (0.05 M, pH 6.5). The absorbance of samples were measured at 476 (betacyanin), 535 (betaxanthin), and 600 (correction) nm with a phosphate buffer used as a blank. The absorbances of betanin and vulgaxanthin-I were calculated using Equations (2)–(4):

(2) $$x=1.095\times \left(a-c\right)$$

(3) $$y=b-z-\frac{x}{3.1}$$

(4) z=a−x

*a*: absorbance at 538 nm
*b*: absorbance at 476 nm
*c*: absorbance at 600 nm
*x*: absorbance of betanin corrected for colored impurities
*y*: absorbance of vulgaxanthin-I corrected for colored impurities
*z*: absorbance of impurities

Betanin and vulgaxanthin-I concentrations in beetroot samples were calculated using Equation (5):

(5) $$BC\left[mg/L\right]=\frac{A\times DF\times MW\times 1000}{EC\times L}$$

*A*: absorbance of betanin corrected for colored impurities (*x*) or absorbance of vulgaxanthin-I corrected for colored impurities (*y*)
*DF*: dilution factor and the pathlength of the 1 cm cuvette.
*MW*: molecular weights of the representative compounds betanin (550 g/mol)and vulgaxanthin-I (308 g/mol)
*EC*: extinction coefficients of the representative compounds betanin (60,000 L/mol·cm) and vulgaxanthin-I (48,000 L/mol·cm)

The betacyanins (BC) content was expressed as mg betanin equivalents per 100 g of dry solid sample (mg_BE_/100 g_dry solid_), and the betaxanthins (BX) content was expressed as mg vulgaxanthin-I equivalents per 100 g of dry solid sample (mg_VE_/100 g_dry solid_).

- Total phenols

Methanol was used for the sample extraction and the Folin-Ciocalteu method was performed for total phenols (TP) analysis, according to the method described by Igual et al. [33]. The samples were mixed with methanol, and HCl 5N, NaF 2 mM Extract was mixed with Folin-Ciocalteu reagent and Na_2_CO_3_ and stored in the dark for 120 min. The samples absorbance (UV-3100PC, VWR, Radnor, Philadelphia, USA) at 765 nm was measured and expressed as mg gallic acid/100 g of dry solid sample (mg GAE/100 g_dry solid_).

- Antioxidant capacity

The antioxidant capacity (AC) was based on the DPPH method, as described previously by Igual et al. [33]. Samples were mixed with methanol. 0.1 mL of extract was mixed with 3.9 mL DPPH (0.030 g/L) and stored for 5 min. The samples’ absorbance was measured at 515 nm and expressed as milligram Trolox equivalents (TE) per 100 g of dry solid sample (mg TE/100 g_dry solid_).

### 2.6. Statistical Analysis

Statgraphics Centurion XVII Software, version 17.2.04 was used for Analysis of Variance (ANOVA), with a confidence level of 95% (*p* < 0.05). ANOVA was applied to evaluate the differences among mixtures, pellet and expanded snacks. The method used to discriminate between means was Fisher’s least significant difference procedure. Pearson correlation analysis (Statgraphics Centurion XVII), with a 95% significance level was conducted to establish the relationships among the studied parameters.

## 3. Results and Discussion

### 3.1. Microwaving Expansion Kinetics

**Figure 2:**
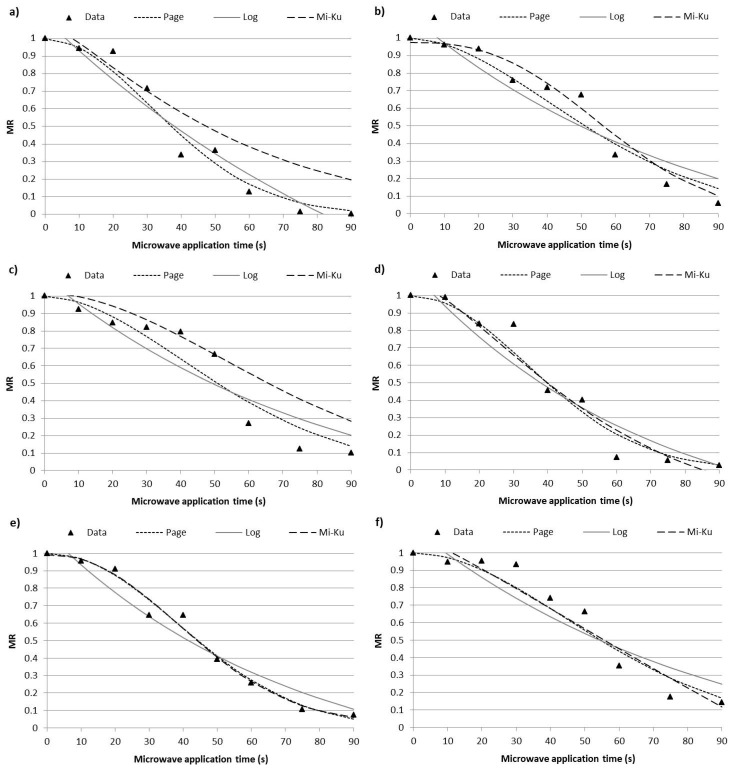
Microwaving expansion kinetics of corn pellets with a different water and beetroot content of its mixtures (**a**) Control snack with 25% water content in mixture, (**b)** Control snack with 30% water content in mixture; (**c**) Snack with 25% water content and 5% beetroot content in mixture, (**d**) Snack with 30% water content and 5% beetroot content in mixture, (**e**) Snack with 25% water content and 10% beetroot content in mixture, and (**f**) Snack with 30% water content and 10% beetroot content in mixture, adjusted to Page, Logarithmic (Log), and Midilli–Kucuk (Mi-ku) models.

Microwave dehydration curves of pellets with different moisture (25% and 30%) and % beetroot byproduct (0, 5, and 10) were obtained by plotting the moisture ratio (MR) vs. time. The experimental data were fitted to the Page, Logarithmic, and Midilli-Kucuk models. Figure 2 shows the MR microwave process experimental data at different studied times. The kinetic parameters and the accuracy of the fit determined for the three models are presented in Table 1. These models fit well with the experimental data, as seen from the adjusted regression coefficient (R^2^) and the root mean square error (RMSE) values (Table 1). The best fit (higher R^2^) was the Page model for all samples except C30. In this sample, the Midilli-Kucuk model presented the best fit. Therefore, in general terms, the Page model provided the best fit with the experimental data.

In Table 1, the model parameters for each mathematic model are exhibited. For the Page models, the parameter *k* could be related with the diffusion coefficient and the geometry of the sample. The trend of the values indicates a higher water diffusion coefficient at 25% of moisture without beetroot byproduct and an inversely proportional trend to the water content; a similar effect was shown in starch-gluten-water mixtures heated by microwave [34]. The incorporation of beetroot byproducts in the mixtures reduces the values of *k*. The parameter *n* is associated with the type of diffusion phenomenon (*n* > 1 for super-diffusion and *n* < 1 for sub-diffusion). According to Simpson et al. [35], this exponent should be related with the microstructure; for this reason, a fluctuation inversely proportional to the water content of the mixtures was observed. The addition of beetroot byproduct increased parameter *n* in general terms. In all samples, parameter *n* was higher than the unit due to the high heating velocity during the microwave process.

**Figure 3:**
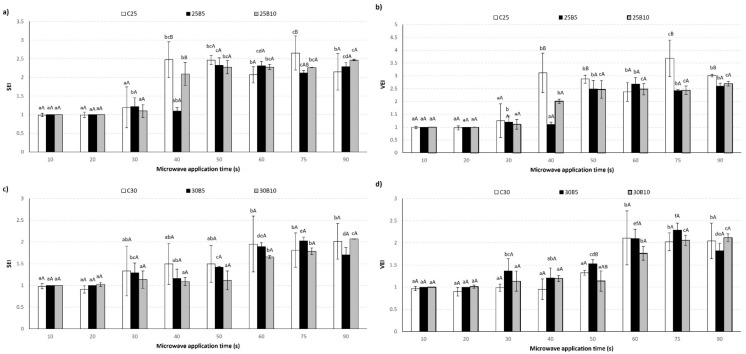
Evolution of SEI (**a**,**c**) and VEI (**b**,**d**) of the pellets as a function of the processing time for studied samples (C25: Control snack with 25% water content in mixture, C30: Control snack with 30% water content in mixture; C25B5: Snack with 25% water content and 5% beetroot content in mixture, C25B10: Snack with 25% water content and 10% beetroot content in mixture, C30B5: Snack with 30% water content and 5% beetroot content in mixture, and C30B10: Snack with 30% water content and 10% beetroot content in mixture). Different letters on the bars a, b, c, d, e, f represent significant differences (*p* < 0.05) by microwave expanded application time, and A, B represent significant differences (*p* < 0.05) by water content.

**Figure 4:**
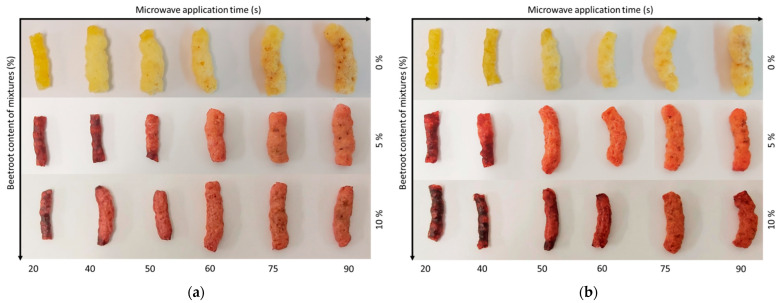
(**a**) Evolution of the appearance of snack with 25% of water content as a function of the processing time for the beetroot content of its mixtures. (**b**) Evolution of the appearance of snack with 30% of water content as a function of the processing time for beetroot content of its mixtures.

Figure 3 and Figure 4 show the evolution of the sectional expansion index (SEI) and volumetric expansion index (VEI) of the pellets as a function of the microwave application time and the appearance of pellets as a function of the microwave application time, respectively. At the beginning of the process (10 to 30 s), microwave energy heated the matrix through the vibration of water molecules, and the temperature of the pellets increased progressively; however, these pellets did not show significant (*p* > 0.05) changes in SEI and VEI values. The control pellet from the mixture with 25% moisture expanded at 40 s, while the 30% moisture in mixture expanded at 60 s.

The addition of beetroot byproduct in mixtures increased the expansion time compared with control, this behavior was primarily observed in SEI. This finding can be related to the fiber content of the beetroot, which limits the expansion of the snack by interaction with the water molecules present in the matrix [36,37,38]. The competition for available water between the fiber and starch leads to a slowdown in the starch gelatinization process, which also explains the differences observed [39].

The pellets with 30% of water content showed lower SEI and VEI values at each time, while, in contrast, pellets with 25% water content exhibited a higher expansion index in all the application times evaluated.

The trends observed in the SEI and VEI values are also reflected in Figure 4. It can be observed that in the last treatment times (75 s for samples with 25% moisture in mixture and 90 s for samples with 30% moisture in mixture), the samples burned and both presented brown colors, with the control samples and those containing beetroot byproduct in their formulation. In agreement with other studies [20], if microwave heating is terminated at the appropriate time, the matrix reverts to a glassy state and the foamed air cells are retained, generating a crispy or crunchy texture. Conversely, the matrix might burn if microwave heating is not terminated in a timely manner, as occurs in these indicated times.

According to a microwave expansion kinetics study, the best time was obtained according to dehydration kinetics (stability), expansion indexes (high), and the absence of burning. The selected times for C25, 25B5 and 25B10 were 50 s, 60 s and 60 s, respectively. In case of C30, 30B5 and 30B10 they were 60 s, 60 s and 75 s, respectively.

### 3.2. Snacks Characterization

Once the microwave treatment times have been defined, it is necessary to study the physicochemical properties affected by water and by beetroot byproduct content in the mixtures, as well as the monitoring of the main bioactive compounds of beetroot that provide functional value to the snack.

#### 3.2.1. Physicochemical Properties

**Figure 5:**
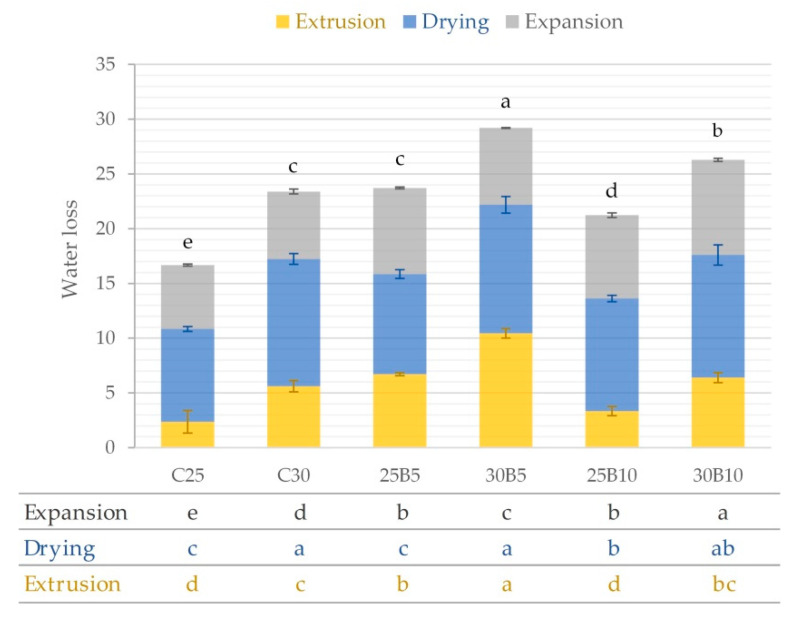
Mean values (and standard deviations) of water loss due to extrusion, drying, and expansion for each snack. The table contains the letters representing the homogeneous groups established by ANOVA (*p* < 0.05) for water losses at each stage of production.

Figure 5 shows water loss due to extrusion, drying, and expansion for each snack. The highest water loss was suffered by samples during the drying step. Samples with 25% water content in mixtures lost less water during extrusion than samples with 30% water content in mixtures comparing C25-C30, 25B5-30B5, and 25B10-30B10. During the microwave expansion process, the samples containing the highest beetroot byproduct content lost significantly (*p* < 0.05) more water than the control samples. The beetroot byproduct provides snack fiber, so there is a greater amount of water to be absorbed by this component and, consequently, the loss of water during microwave expansion will be greater. Therefore, expansion will be smaller, as besides the higher water loss, insoluble fibers are also biopolymers which are already slightly extendable, unlike starch [40]. This trend was also observed in 2G snacks during the expansion of corn with nettle, which also contained a large amount of fiber in its composition [41]. The largest total water loss occurred in 30B5 and 30B10, with significant differences with the other samples (*p* < 0.05). These samples contained the highest amount of water in the initial mixture and also contain beetroot byproduct in their formulation.

Table 2 shows the water content and hygroscopicity of pellets and extrudates, pellet water activity and characteristic parameters of extrudates. The water content of pellets ranged from 9.2% to 10%, similar to other works [19]. According to some studies, mixtures with approximately 20–30% moisture are subjected to produce pellets with approximately 9–15% moisture equally dispersed throughout the pellet’s volume. With this moisture, the final product has shown better expansion indexes, light structure, and crispy texture [42,43].

The values of a_w_^P^ of samples with 25% of water content in mixtures were significantly (*p* < 0.05) higher than a_w_^P^ of samples with 30% of water content in mixtures for pellets with 0% and 5% of beetroot byproduct. However, pellets with 10% of beetroot byproduct did not present significant (*p* > 0.05) differences for % of water content in mixtures.

Hygroscopicity is a parameter related to the gaining of water from the sample during storage in high relative humidity. The pellets containing beetroot byproduct were significantly (*p* < 0.05) more hygroscopic due to their higher fiber content, and, therefore, would be less stable during storage. However, after microwave expansion, the hygroscopity of the snacks were similar, without showing significant differences (*p* > 0.05). No significant (*p* > 0.05) effect was observed for the water content in the mixtures (25% or 30%) on the water content of the microwave expanded product. However, the addition of beetroot byproduct in the percentages studied decreased (*p* < 0.05) the water content of the snacks significantly.

SEI, ρ_b_ and ε are parameters that reflect the expansion of the expanded snacks. The trend of all three was similar. The addition of beetroot byproduct reduced SEI significantly (*p* < 0.05) and ε, and increased significantly (*p* < 0.05) ρ_b_, therefore these samples were denser, less porous, and less expanded. This behavior was due to the higher fiber content of the snacks enriched with beetroot byproduct, as observed by other authors [36,37,38,39], and the addition of fiber limits the expansion of the snack. During extrusion, the fibers create a network that affects the distribution of water in the matrix, modifying the extension characteristics because fiber and starch compete for water, and this leads to a delay in the gelatinization of starch, therefore, to reduced expansion [44]. A significant effect (*p* < 0.05) of the water content of the initial mixture was also observed. The most humid mixture (30%) presented lower values of SEI and ε, and higher values of ρ_b_. There is a significant positive correlation between a_w_^P^ and SEI (0.8095, *p* < 0.05) according to the Pearson analysis. Pellets with higher water activity allow for the obtaining of more expanded snacks.

The hydration properties were affected by the addition of beetroot byproduct and by the water content of the mixture. When the water content of the mixture was higher, WAI increased while WSI decreased. On the other hand, when beetroot byproduct was added, WAI decreased while WSI increased. According to different authors [45,46,47,48], these two indices inform about biopolymers’ physicochemical changes because of extrusion processing. WAI indicates the amount of water immobilized by the extrudate [49], whereas WSI indicates the amount of small molecules solubilized in water causing molecular damage in the process [50]. Therefore, the use of 30% water content in the mixture to extrusion reduces the risk of possible molecular damage by molecules solubilized in water compared with the use of 25%. The SWE of corn samples without beetroot byproduct and with 25% water content in mixture was similar to the SWE of 2G corn snacks [27,39]. Samples with 25% water content in mixture increased SWE when beetroot byproduct was added, probably for the hydration of the fiber. However, samples with 30% water content in the mixture not showed a clear trend by effect of beetroot byproduct addition. Samples with 30% water content in mixture and without beetroot byproduct presented significant higher SWE than the same % of beetroot byproduct but with 25% water content in mixture.

A correlation analysis was applied in order to better explain the relationship between water losses suffered by samples during each process step and typical extrudate parameters. Extrusion water loss was positively correlated with ρ_b_ (0.7222, *p* < 0.05) and negatively correlated with ε (−0.8320, *p* < 0.05). Therefore, the higher the extrusion water loss, the denser and less porous the snacks. This behavior was observed in other works with 2G snacks [39,41]. Loss of water during the microwave expansion process was negatively correlated with x_w_^E^ (−0.7528, *p* < 0.05). The higher the expansion water loss, the lower the water content in the snacks.

The texture of snacks is one of the most interesting properties [51]. Hardness and crispness will be addressed in this study through the parameters of crispness work (W_c_), average specific force of structural ruptures (F_s_), average puncturing force (F_p_), spatial frequency of structural ruptures (N_sr_), and the number of peaks (N_0_), as shown in Table 3. W_c_ is related to the sensory criterion of fracturability [52], described as the force applied on the first bite to break the sample [53]. F_s_ and F_p_ are properties usually associated with the sensory attribute of hardness during chewing (force required to crush a substance among the molar teeth) [54]. N_sr_ indicates the number of fracture episodes that occur during puncture [52], and N_0_ is the number of fractures during the puncture test [55]. Comparing the texture values of the control samples (0% beetroot byproduct) with the values of the 2G snacks from other works [27,30,39,41], the differences between the types of 3G-2G snacks can be observed. 3G snacks are harder and less crisp than 2G snacks, probably because they are denser and less porous, and there is less air in the pores of their structure. Almost no significant effect of % water content in mixtures on snack textures was observed. They only showed significant differences in the N_sr_ parameter in the samples enriched with beetroot byproduct. The higher the water content in the mixtures with beetroot byproduct, the higher the fracture episodes in the puncture test. In terms of texture, overall, the hardest samples were those enriched with 10% beetroot byproduct for both snacks from mixtures with 25% or 30% water content.

By establishing relationships among texture parameters, physicochemical parameters, and water losses during each stage of the process using Pearson correlations, positive and significant (*p* < 0.05) correlations were observed between F_s_ and ρ_b_ (0.7287), and F_s_ and expansion water loss (0.7978). Therefore, higher water losses during microwave expansion result in harder snacks. It is likely that the violent and rapid water loss during microwave heating causes the harder structure. In addition, denser snacks are harder, as shown by other work on 2G snacks [39]. Many studies have reached the same results by relating hardness to bulk density [56]. N_sr_ was significantly (*p* < 0.05) and negatively correlated with ε. The higher the porosity, the lower the number of fractures during the test, indicating that the pores are probably larger and therefore there are fewer ruptures as the probe advances.

**Figure 6:**
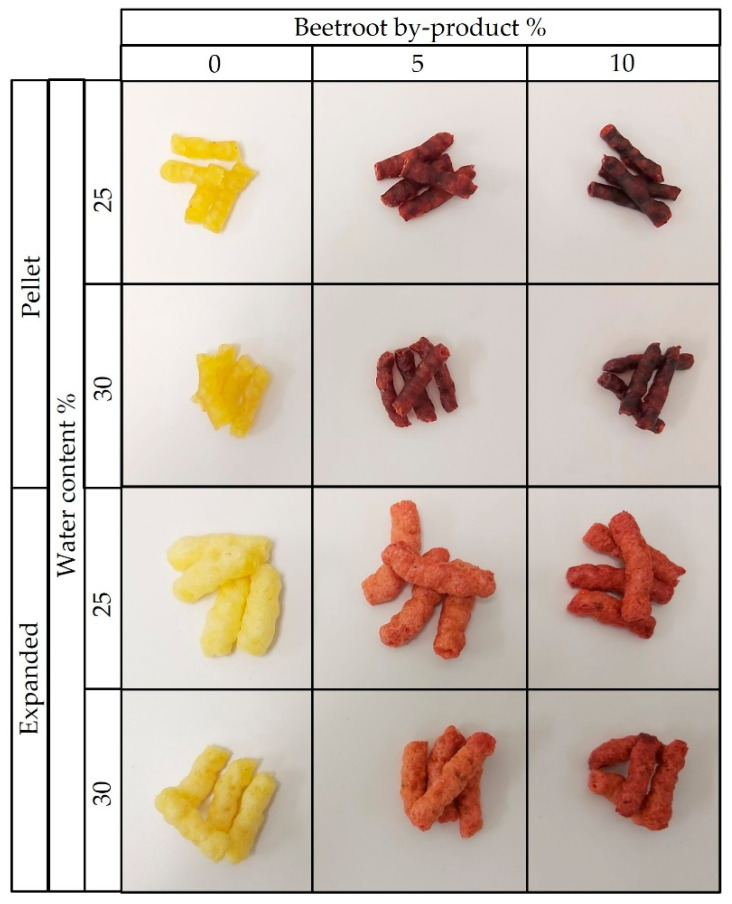
The appearance of pellets after extrusion and drying and expanded products by microwaves. They are shown at two moisture levels (25% and 30%) and three percentages of beetroot byproduct (0, 5 and 10).

Table 4 shows the color coordinates and the differences between the color of the mixtures and the expanded snacks, as well as the differences between the pellets and the expanded snacks. The incorporation of beetroot byproduct in the mixtures to obtain the 3G snacks caused significant changes in the color coordinates of the expanded samples. a* increased, while L*, b*, C* and h* decreased, obtaining darker and reddish samples. This behavior was more pronounced when higher concentrations of beetroot byproduct were used in the mixtures. Comparing the snacks with the same levels of beetroot byproduct but different water content in the mixtures, marked differences in L*, b*, C*, and h* were found only in the snacks containing 10% beetroot byproduct. The trends described at the quantitative level can be seen in Figure 6, which shows the appearance of the expanded snacks at the bottom. It also includes the pellets from which the expanded snacks were obtained in the upper part. The differences in appreciable color in Figure 2 between each pellet with its expanded pellet are reflected in Table 4 as ΔE_2_. For both the 25% and 30% water content mixtures, the snack with 5% beetroot byproduct in its composition showed the highest ΔE_1_ and ΔE_2_. ΔE_2_ was higher than ΔE_1_, probably because of the starch structure in the pellet, which is gelatinized.

#### 3.2.2. Bioactive Compounds

One of the hypotheses of this work is that the use of beetroot byproduct increases the functional value of 3G snacks. As can be seen in Table 5, the incorporation of beetroot byproduct in the mixtures to be extruded at the two studied moisture levels in the mixtures increased AC, TP, and above all betalains (BC and BX) significantly (*p* < 0.05). This effect was observed in the mixtures and also in the final snacks that were submitted to extrusion plus drying plus microwave expansion. Betalains are the characteristic pigments of beetroot [57,58], and according to these results, they are in the mixtures and in the 3G snacks obtained. The results obtained for the pigments of the samples and the color coordinates were correlated by means of Pearson coefficients, and in all cases significant (*p* < 0.05) correlations were obtained. Both pigments showed negative correlations with L*, b*, C*, and h* and positive correlations with a*. This is a logical trend if it is taken into account that the pigments studied give reddish tones. These correlation coefficient values between BC and L*, a*, b*, C*, and h* were −0.8536, 0.7688, −0.9046, −0.9351, and −0.8415, respectively, and for BX and L*, a*, b*, C*, and h* they were −0.8025, 0.7289, −0.8645, −0.9020, and −0.8005, respectively. There was a significant effect of the water content of the mixture on BC content in mixtures and the expanded snacks. The BC content was significantly lower in samples with 30% water content in mixtures than samples with 25% for the same level of beetroot byproduct content. This behaviour can be explained in part by the degradation of the pigments of red beetroot due to the increase in water content, as was observed by other authors [59]. Betalains decreased as the samples were extruded, dried and expanded to obtain the expanded snacks.

Table 5 also shows the significant (*p* < 0.05) increase in TP and AC when incorporating beetroot byproduct in the mixtures, and it is also maintained after obtaining the expanded product. TP and AC decreased as the samples were extruded, dried and expanded to obtain the expanded snacks. This fact was observed by other authors when adding beetroot to Asian noodles [60]. However, the effect of the % level of beetroot byproduct is only significant (*p* < 0.05) in TP for mixtures and in AC for expanded snacks. The processes studied in this work probably affects TP without a proportional relationship to their initial content.

In order to explain the influence of BC, BX, and TP quantified in this study on the antioxidant capacity of the samples, correlational statistical analyses were performed. BX played a major role in the antioxidant capacity (0.8997, *p* < 0.05), followed by BC (0.8936, *p* < 0.05) and finally TP (0.7378, *p* < 0.05).

**Figure 7:**
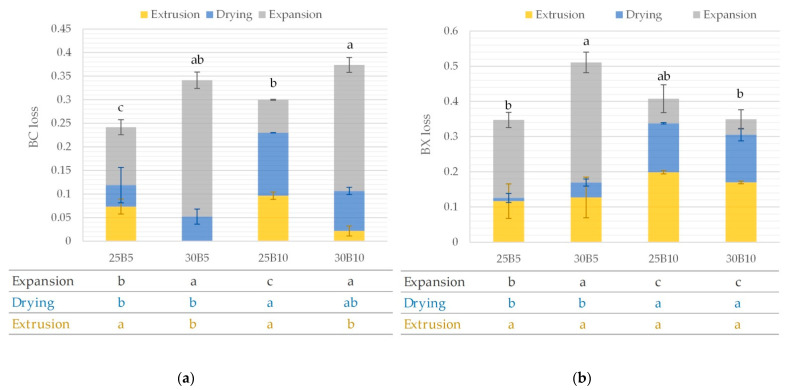
Mean values (and standard deviations) of (**a**) betacyanins (BC) and (**b**) betaxanthins (BX) loss due to extrusion, drying, and expansion for each snack. The table contains the letters representing the homogeneous groups established by ANOVA (*p* < 0.05) for betalain losses at each stage of production.

The different stages that take place until a 3G snack is obtained involve the degradation of the initial bioactive compounds in the mixture. Figure 7 shows the variations of the betalains of the beetroot byproduct snacks at the different steps of the process (extrusion, drying, and expansion). The loss of BC or BX (ΔMi), referring to the mixture content, were calculated according to Equations (6)–(8).

(6) $$∆M_{i}^{ET}=\frac{\left(M_{i}^{M}-M_{i}^{ET}\right)}{M_{i}^{M}}$$

(7) $$∆M_{i}^{D}=\frac{\left(M_{i}^{ET}-M_{i}^{D}\right)}{M_{i}^{M}}$$

(8) $$∆M_{i}^{EP}=\frac{\left(M_{i}^{D}-M_{i}^{EP}\right)}{M_{i}^{M}}$$

where: *Mi*: mass of *BC* or *BX* in the sample referred to dried solid sample and superscripts: *M*: mixtures, *ET*: extrusion, *D*: drying, *EP*: expansion.

In Figure 7 it can be observed that in an overall view, there are greater losses in BX than in BC. This trend was observed by other authors who also described higher losses in BX than in BC in cooking processes [61]. Therefore, BX could be more vulnerable to losses during processing. The degradation of betalains corresponds mainly to isomerization, hydrolysis, decarboxylation reactions, etc. [62]. The factors primarily causing them are temperature, contact with oxygen and light, water activity, pH, etc. [62]. Figure 7a shows significantly (*p* < 0.05) higher BC losses in the snacks containing 30% water content in the mixtures compared to the samples containing 25% water in the mixture. It is likely that a matrix with more water in the initial mixtures increases the degradation of BC. The presence of a higher water content, if available, increases the solubility of oxygen and the mobility of molecules that can negatively impact the betalain content [61]. In addition, the microwave expansion stage causes the greatest contribution to these losses. Betalains are water-soluble compounds [63], and, it is probable that a higher availability of water moving through the microwave energy (temperature increase) with the dissolved pigments promotes to a higher degradation of these compounds. However, snacks from 25% water content mixes showed significantly higher BC losses than those from 30% water content mixes in the extrusion process.

Figure 7b includes the mean values and standard deviation of BX losses due to the extrusion, drying, and expansion for each snack. The concentration of the beetroot byproduct or the water content of the mixture does not show a significant (*p* > 0.05) impact on the BX loss during the extrusion stage. This process results in a loss of 25–50% of the total content of these compounds. In drying, BX losses are significantly (*p* < 0.05) higher in samples with 10% beetroot byproduct than in those with 5% beetroot byproduct. The opposite is true for the expansion stage, where the higher the beetroot concentration, the lower the BX losses. Taking into account the results of Figure 7, the formulation with 25% water content in the mixture and 5% beetroot byproduct suffers less loss of betalains during production. Finally, the expanded snack with a high betalains content (Table 5) was 25B10 (25% water content and 10% beetroot byproduct in mixture). In addition, this snack showed the highest antioxidant capacity values.

## 4. Conclusions

The use of beetroot byproducts to produce third-generation value-added snacks is feasible according to the results obtained. The microwave expansion kinetics study determined the appropriate time to expand the formulations studied. Samples with higher water content in the mixtures needed more expansion time. Similarly, the snacks with beetroot byproduct needed more expansion time compared to the corn-only snacks. In terms of expansion, all samples presented acceptable values, although samples with 25% water in the mixtures showed better results. Furthermore, these snacks showed more crunchiness and less hardness. On the other hand, the addition of beetroot byproduct provided additional functional value to the snacks, increasing their content of betalains and phenols. The betalains contained in the beetroot byproduct were presented in the expanded snacks and increased the antioxidant capacity of the snacks. With this study, it can be recommended to use 25% water content and 10% beetroot byproduct in corn mixture to obtain a third-generation snack with added value.

This work shows that the reuse of beetroot byproduct adds value to 3G snacks and encourages sustainable production, promoting the circular economy in the food industry.

## Figures and Tables

**Table 1:** Values of the microwaving expansion kinetic parameters obtained for pellets (C25: Control snack with 25% water content in mixture, C30: Control snack with 30% water content in mixture; C25B5: Snack with 25% water content and 5% beetroot content in mixture, C25B10: Snack with 25% water content and 10% beetroot content in mixture, C30B5: Snack with 30% water content and 5% beetroot content in mixture, and C30B10: Snack with 30% water content and 10% beetroot content in mixture) when the Page, Logarithmic, and Midilli–Kucuk models were used to fit the experimental data.

Sample	Model			
		Page	Logarithmic	Midilli–Kucuk
C25	Model constants	k: 0.00062 n: 1.93984	a: 2.2581 k: 0.0082 c: −1.150	a: 1.0939 k: 0.0069 n: 1.2271 b: −0.0029
	Adj. R^2^	96.36	91.52	91.41
	RMSE	0.078	0.119	0.120
C30	Model constants	k: 0.00054 n: 1.81839	a: 1.4349 k: 0.0115 c: −0.3080	a: 0.9752 k: 0.00002 n: 2.6097 b: −0.0006
	Adj. R^2^	93.99	84.15	95.93
	RMSE	0.086	0.140	0.071
25B5	Model constants	k: 0.00053 n: 1.82760	a: 1.3991 k: 0.0116 c: −0.2898	a: 1.0185 k: 0.00028 n: 1.8714 b: −0.0026
	Adj. R^2^	90.36	78.79	89.15
	RMSE	0.110	0.163	0.116
25B10	Model constants	k: 0.00044 n: 2.00154	a: 1.5595 k: 0.0140 c: −0.4164	a: 1.0831 k: 0.0140 n: 1.4746 b: −0.0018
	Adj. R^2^	95.54	87.83	91.46
	RMSE	0.086	0.1424	0.119
30B5	Model constants	k: 0.00028 n: 2.05887	a: 1.4313 k: 0.0135 c: −0.3159	a: 0.9927 k: 0.0002 n: 2.1548 b: 0.0003
	Adj. R^2^	98.22	91.93	97.55
	RMSE	0.048	0.1029	0.057
30B10	Model constants	k: 0.00036 n: 1.89348	a: 1.4084 k: 0.0111 c: −0.2683	a: 1.1026 k: 0.0016 n: 1.3214 b: −0.0054
	Adj. R^2^	93.43	80.02	88.25
	RMSE	0.089	0.1549	0.119

a, b, c and k: Drying constants. n: Drying exponent. Adjusted regression coefficient (Adj. R^2^) and root mean square error (RMSE) values.

**Table 2:** Mean values (and standard deviations) of pellet water content ($x_{w}^{P}$), pellet water activity ($a_{w}^{P}$), pellet hygroscopicity (Hy^P^), extrudate water content ($x_{w}^{E}$), extrudate hygroscopicity (Hy^E^), and section expansion index (SEI), bulk density (ρ_b_), porosity (ε) water absorption index (WAI), water solubility index (WSI), and swelling index (SWE) of expanded products.

**Beetroot** **%**	$x_{w}^{P}$ $\left(g_{w}/100\;g\right)$	$a_{w}^{P}$	$\begin{array}{c} Hy^{P}\\ \left(g_{w}/100\;g_{dry\;solid}\right) \end{array}$	$x_{w}^{E}\;\left(g_{w}/100\;g\right)$	$\begin{array}{c} Hy^{E}\\ \left(g_{w}/100\;g_{dry\;solid}\right) \end{array}$	SEI	ρ_b_ (g/cm^3^)	ε (%)	WAI	WSI (%)	SWE (mL_swollen_/g_dry solid_)
	25 % **Water Content in Mixture**										
0	9.4 (0.2) ^aA^	0.565 (0.002) ^aA^	13.165 (0.113) ^aC^	4.1 (0.7) ^aA^	23 (2) ^aA^	7.2 (0.6) ^aA^	0.209 (0.002) ^bB^	70 (1.3) ^aA^	7.3 (0.4) ^bA^	10.6 (0.5) ^aC^	2.36 (0.04) ^bC^
5	9.24 (0.08) ^aA^	0.568 (0.003) ^aA^	14.5 (0.4) ^bB^	1.4 (0.2) ^aB^	24 (2) ^aA^	7.75 (0.9) ^aA^	0.27 (0.02) ^bA^	64.5 (0.6) ^aB^	6.26 (0.09) ^bB^	14.23 (0.13) ^aA^	3.5525 (0.0003) ^bB^
10	9.4 (0.2) ^bA^	0.543 (0.008) ^aB^	15.8 (0.2) ^aA^	1.7 (0.7) ^aB^	24 (2) ^aA^	6.5 (0.4) ^aB^	0.246 (0.013) ^bA^	65.8 (1.4) ^aB^	5.7 (0.2) ^bC^	13.2 (0.4) ^aB^	5.230 (0.097) ^aA^
	**30% Water Content in Mixture**										
0	9.6 (0.2) ^aB^	0.5465 (0.0106) ^bA^	13.1 (0.4) ^aB^	3.8 (0.7) ^aA^	20.7 (1.4) ^aB^	6.3 (0.3) ^bA^	0.265 (0.007) ^aB^	65 (2) ^aA^	8.821 (0.006) ^aA^	2.54 (0.03) ^bB^	5.412 (0.206) ^aA^
5	9.21 (0.04) ^aC^	0.5365 (0.0007) ^bA^	15.3 (0.2) ^aA^	2.2 (0.5) ^aB^	23.1 (0.7) ^aA^	5.5 (0.4) ^bB^	0.35 (0.05) ^aA^	55 (4) ^bB^	7.70 (0.12) ^aB^	6.8 (0.2) ^bA^	4.54 (0.04) ^aB^
10	10.00 (0.13) ^aA^	0.545 (0.002) ^aA^	14.82 (0.06) ^bA^	1.3 (0.9) ^aB^	22.15 (1.08) ^aAB^	4.9 (0.5) ^bB^	0.335 (0.012) ^aA^	58 (2) ^bB^	6.5 (0.4) ^aC^	6.6 (0.5) ^bA^	5.3 (0.2) ^aA^

Different letters in lowercase superscripts represent significant differences (*p* < 0.05) by water content in mixture % for each sample with the same beetroot %, and uppercase superscripts represent significant differences (*p* < 0.05) by beetroot % for each sample with the same water content in mixtures.

**Table 3:** Mean values (and standard deviations) of texture parameters of expanded product: crispness work (W_c_), average specific force of structural ruptures (F_s_), average puncturing force (F_p_), spatial frequency of structural ruptures (N_sr_), and number of peaks (N_0_).

Beetroot %	$W_{c}\;(N\cdot \mathrm{mm})$	$F_{s}\;(N)$	$F_{p}\;(N)$	$N_{sr}\;({\mathrm{mm}}^{-1})$	$N_{0}$
**25% Water Content in Mixture**					
0	0.61 (0.04) ^aB^	4.60 (0.13) ^bB^	4.2 (0.8) ^aB^	7.4 (0.7) ^aA^	63 (8) ^aA^
5	0.71 (0.05) ^aB^	5.5 (0.4) ^aA^	4.5 (0.2) ^aB^	7.8 (0.3) ^bA^	54 (6) ^aAB^
10	0.9 (0.2) ^aA^	6.07 (1.02) ^aA^	6.0 (1.3) ^aA^	7.1 (0.7) ^bA^	50 (8) ^aB^
**30% Water Content in Mixture**					
0	0.69 (0.07) ^aA^	5.3 (0.5) ^aB^	4.5 (0.3) ^aB^	7.2 (1.3) ^aB^	50 (8) ^bA^
5	0.7 (0.2) ^aA^	5.8 (1.3) ^aB^	5.7 (1.2) ^aAB^	8.8 (0.4) ^aA^	48 (6) ^aA^
10	0.78 (0.12) ^aA^	7294 (1108) ^aA^	6.22 (1.03) ^aA^	9.3 (0.6) ^aA^	49 (9) ^aA^

Different letters in lowercase superscripts represent significant differences (*p* < 0.05) by water content in mixture % for each sample with the same beetroot %, and uppercase superscripts represent significant differences (*p* < 0.05) by beetroot % for each sample with the same water content in mixtures.

**Table 4:** Mean values (and standard deviations) of color coordinates (L*, a*, b*, C*, and h*) and total color differences (ΔE_1_ and ΔE_2_) of the finished expanded product. ΔE_1_: total color difference between the mixture before extrusion and the finished expanded product. ΔE_2_: total color differences between the dry pellet and the finished expanded product.

Beetroot %	L*	a*	b*	C*	h*	ΔE_1_	ΔE_2_
**25% Water Content in Mixture**							
0	77.00 (0.13) ^aA^	5.38 (0.04) ^aC^	41.2 (0.4) ^aA^	41.6 (0.4) ^aA^	82.57 (0.02) ^aA^	3.9 (0.4) ^bC^	8.95 (0.09) ^aC^
5	56.5 (0.8) ^aB^	23.9 (0.4) ^aB^	26.1 (0.5) ^aB^	35.4 (0.7) ^aB^	47.44 (0.09) ^bB^	16.9 (0.4) ^aA^	20.2 (0.5) ^aA^
10	41 (2) ^bC^	24.5 (0.4) ^aA^	17.2 (0.7) ^bC^	29.9 (0.7) ^bC^	35.0 (0.7) ^bC^	10.5 (2) ^aB^	13 (2) ^aB^
**30% Water Content in Mixture**							
0	76.8 (0.2) ^aA^	5.26 (0.07) ^aC^	40.10 (0.09) ^bA^	40.44 (0.09) ^bA^	82.53 (0.09) ^aA^	4.89 (0.09) ^aC^	5.9 (0.2) ^bC^
5	55.7 (0.8) ^aB^	22.780 (0.104) ^bB^	26.405 (0.006) ^aB^	34.87 (0.07) ^aB^	49.22 (0.12) ^aB^	11.8 (0.6) ^bA^	19.9 (0.7) ^aA^
10	51.4 (0.8) ^aC^	24.09 (0.09) ^aA^	23.00 (0.13) ^aC^	33.30 (0.15) ^aC^	43.68 (0.07) ^aC^	8.2 (0.6) ^bB^	13.8 (0.6) ^aB^

Different letters in lowercase superscripts represent significant differences (*p* < 0.05) by water content in mixture % for each sample with the same beetroot %, and uppercase superscripts represent significant differences (*p* < 0.05) by beetroot % for each sample with the same water content in mixtures.

**Table 5:** Mean value (and standard deviation) of betacyanins (BC, mg_BE_/100g_dry solid_), betaxanthins (BX, mg_VE_/100g_dry solid_) total phenol (TP, mg_GAE_/100g_dry solid_) content, and antioxidant capacity (AC, mg_TE_/100g_dry solid_) of mixtures and extrudates.

Beetroot %	BC	BX	TP	AC	BC	BX	TP	AC
Mixtures					Expanded Products			
**25% Water Content in Mixture**								
0	-^aC^	-^aC^	33 (2) ^aC^	2.0 (0.4) ^aB^	-^aC^	-^aC^	19.1 (0.8) ^aB^	1.5 (0.3) ^aC^
5	7.48 (0.12) ^aB^	6.9 (0.2) ^aB^	38.7 (1.4) ^bB^	7.79 (0.13) ^bA^	5.67 (0.12) ^aB^	4.64 (0.15) ^aB^	20.2 (1.2) ^aB^	4.22 (0.15) ^aB^
10	11.7 (0.2) ^aA^	12.4 (0.2) ^aA^	45 (2) ^aA^	7.5 (0.7) ^aA^	8.211 (0.012) ^aA^	7.3 (0.5) ^aA^	25 (1) ^aA^	6.4 (0.2) ^aA^
**30% Water Content in Mixture**								
0	-^aC^	-^aC^	34.18 (1.09) ^aC^	3.0 (0.3) ^aB^	-^aC^	-^aC^	20.2 (0.6) ^aB^	1.33 (0.12) ^aC^
5	6.3 (0.2) ^bB^	6.7 (0.2) ^aB^	41.93 (1.09) ^aB^	8 (0.12) ^aA^	4.236 (0.109) ^bB^	3.3 (0.2) ^bB^	19.9 (0.5) ^aB^	4.27 (0.06) ^aB^
10	9.89 (0.12) ^bA^	12.0 (0.4) ^aA^	48 (3) ^aA^	9.5 (0.9) ^aA^	6.19 (0.16) ^bA^	7.8 (0.3) ^aA^	24.45 (1.02) ^aA^	5.2 (0.3) ^bA^

Different letters in superscripts a, b represent significant differences (*p* < 0.05) by water content in mixture % for each sample with the same beetroot %, and A, B, C represent significant differences (*p* < 0.05) by beetroot % for each sample with the same water content in mixtures. “–“: Not detected.

## Data Availability

The data presented in this study are available on request from the corresponding author.
